# Correction: Dhatwalia et al. *Rubus ellipticus* Sm. Fruit Extract Mediated Zinc Oxide Nanoparticles: A Green Approach for Dye Degradation and Biomedical Applications. *Materials* 2022, *15*, 3470

**DOI:** 10.3390/ma15238308

**Published:** 2022-11-23

**Authors:** Jyoti Dhatwalia, Amita Kumari, Ankush Chauhan, Kumari Mansi, Shabnam Thakur, Reena V. Saini, Ishita Guleria, Sohan Lal, Ashwani Kumar, Khalid Mujasam Batoo, Byung Hyune Choi, Amanda-Lee E. Manicum, Rajesh Kumar

**Affiliations:** 1 School of Biological and Environmental Sciences, Faculty of Sciences, Shoolini University of Biotechnology & Management Sciences, Solan 173212, Himachal Pradesh, India; 2 Chettinad Hospital and Research Institute, Chettinad Academy of Research and Education, Kanchipuram 603103, Tamil Nadu, India; 3 Advanced School of Chemical Sciences, Shoolini University of Biotechnology & Management Sciences, Solan 173212, Himachal Pradesh, India; 4 Central Research Laboratory MMIMSR, Department of Biotechnology MMEC, Maharishi Markandeshwar (Deemed to be University), Mullana 133207, Haryana, India; 5 Patanjali Research Institute, Haridwar 249405, Uttarakhand, India; 6 King Abdullah Institute for Nanotechnology, College of Science, King Saud University, Building No. 04, Riyadh 11451, Saudi Arabia; 7 Department of Biomedical Sciences, Inha University College of Medicine, 100 Inha-ro, Incheon 22212, Korea; 8 Department of Chemistry, Faculty of Science, Arcadia Campus, Tshwane University of Technology, Pretoria 0183, South Africa; 9 Department of Physics, Faculty of Physical Sciences, Sardar Vallabhbhai Patel Cluster University, Mandi 175001, Himachal Pradesh, India

## Error in Figure

In the original publication [1], there was a mistake in Figure 5b as published. An incorrect Elemental mapping figure was mistakenly included in the article, which was later noticed. The authors took full responsibility to prompt the amendment, for the inclusion of the correct figure, to represent the science accurately. The corrected Figure 5b appears below. The authors apologize for any inconvenience caused and state that the scientific conclusions are unaffected. This correction was approved by the Academic Editor. The original publication has also been updated.

## Figures and Tables

**Figure 5:**
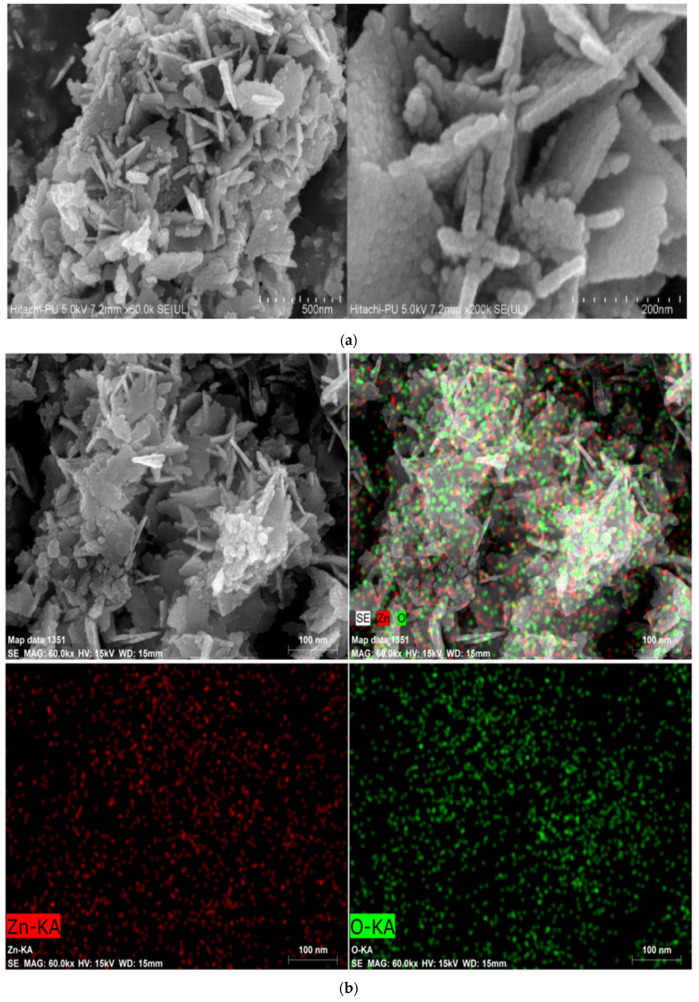
FE-SEM micrographs (**a**) and Elemental mapping (**b**) of ZnO-NPs.
