# Supplementary material for: Rubus ellipticus Sm. Fruit Extract Mediated Zinc Oxide Nanoparticles: A Green Approach for Dye Degradation and Biomedical Applications
Source: Materials (Basel). 2022 Apr 6;15(10):3470. doi: 10.3390/ma15103470 (PMC9147757; doi:10.3390/ma15103470)
Supplement: Supplementary file 1 [file materials-15-03470-s001.zip › materials-1628112-supplementary.pdf]

Supplementary Materials:

Rubus ellipticus Sm. Fruit Extract Mediated Zinc Oxide Nanoparticles: A Green Approach for Dye Degradation and Biomedical Applications

Jyoti Dhatwalia [1], Amita Kumari [1,*], Ankush Chauhan [2], Kumari Mansi [3], Shabnam Thakur [1], Reena V. Saini [4], Ishita Guleria [1], Sohan Lal [1], Ashwani Kumar [5], Khalid Mujasam Batoo [6], Byung Hyune Choi [7], Amanda-Lee E. Manicum [8] and Rajesh Kumar [9,*]

Table S1. Antioxidant activity of fruit extract and ZnO-NPs.

Antioxidant assays	Concentrations	ZnO-NPs	Aqueous fruit extract	Positive control
DPPH (% Inhibition)	20 μg/ml	40.29 ± 0.72	45.55 ± 0.82	50.81 ± 1.02
	40 μg/ml	43.16 ± 0.62	52.79 ± 0.92	57.98 ± 0.54
	60 μg/ml	46.99 ± 0.31	59.97 ± 0.96	64.06 ± 1.13
	80 μg/ml	51.22 ± 0.61	72.46 ± 1.19	74.11 ± 0.92
	100 μg/ml	55.18 ± 0.97	81.69 ± 1.05	79.36 ± 0.51
ABTS (% Inhibition)	20 μg/ml	32.29 ± 0.73	41.18 ± 0.56	46.32 ± 0.92
	40 μg/ml	38.13 ± 0.78	51.13 ± 0.66	55.09 ± 0.74
	60 μg/ml	43.76 ± 0.51	58.63 ± 0.58	62.57 ± 0.68
	80 μg/ml	47.74 ± 0.51	67.08 ± 0.48	74.63 ± 1.22
	100 μg/ml	52.77 ± 0.46	74.91 ± 0.56	77.72 ± 1.47
FRAP (μM/mL $FeSO_4$ equivalents)	20 μg/ml	21.42 ± 0.63	37.09 ± 0.68	40.93 ± 0.63
	40 μg/ml	27.37 ± 0.53	43.06 ± 1.25	48.92 ± 1.66
	60 μg/ml	30.23 ± 0.69	48.03 ± 1.23	55.53 ± 1.25
	80 μg/ml	34.48 ± 0.76	51.98 ± 1.31	59.31 ± 1.34
	100 μg/ml	40.23 ± 1.14	56.94 ± 1.21	64.54 ± 1.59

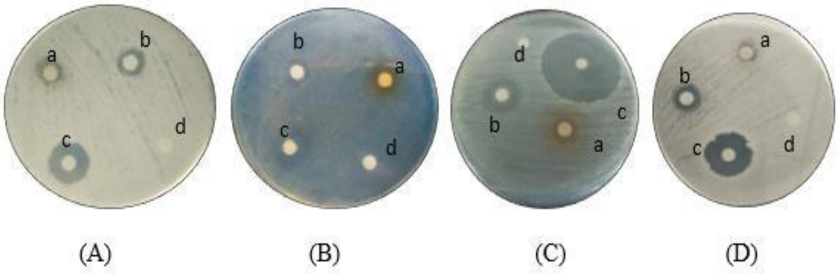

Figure S1. Antibacterial activity of (a- 300 μg; aqueous extract, b- 300 μg; ZnO-NPs, c- 50 μg; Ampicillin, d- negative control (DMSO-10 μl) fruit extract and ZnO-NPs against *E. coli* (A), *P. aeruginosa* (B), *B. subtilis* (C), *S. aureus* (D).

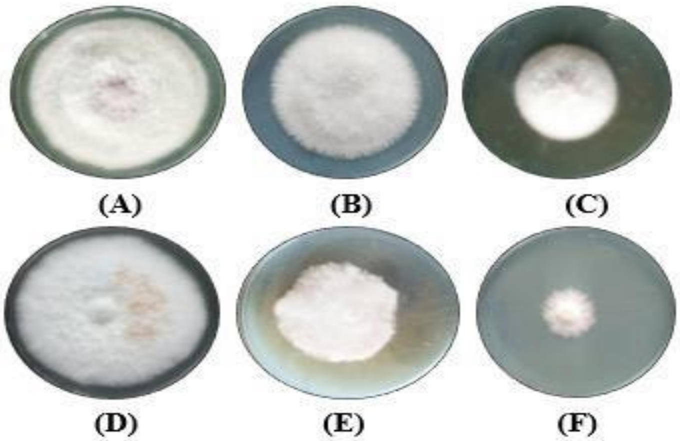

Figure S2. Antifungal activity against *F. oxysporum* and *R. necatrix*; A and D- *F. oxysporum* and *R. necatrix* Control (Without extract and nanoparticles), B and E- Aqueous extract (300 µg), C and F- ZnO-NPs (300 µg).

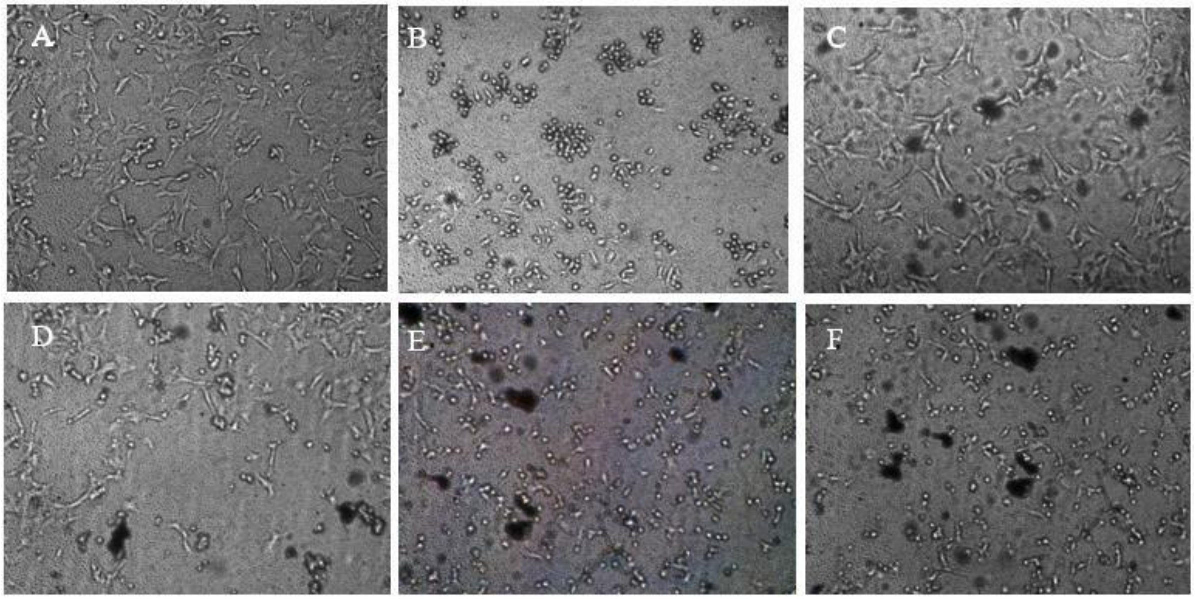

Figure S3. Microscopic images of A549 cell before and after treatment [Cells alone (A); Cells treated with Paclitaxel (B), 25 µg ZnO-NPs (C), 50 µg ZnO-NPs (D), 100 µg ZnO-NPs (E), and 200 µg ZnO-NPs (F)].
